# Optimization of Predictive Performance for the Therapeutic Response Using Iodine Scan-Corrected Serum Thyroglobulin in Patients with Differentiated Thyroid Carcinoma

**DOI:** 10.3390/cancers12020262

**Published:** 2020-01-22

**Authors:** Su Woong Yoo, Md. Sunny Anam Chowdhury, Subin Jeon, Sae-Ryung Kang, Sang-Geon Cho, Jahae Kim, Changho Lee, Young Jae Ryu, Ho-Chun Song, Hee-Seung Bom, Jung-Joon Min, Seong Young Kwon

**Affiliations:** 1 Department of Nuclear Medicine, Chonnam National University Hwasun Hospital, Jeollanam-do 58128, Korea; yoosw.md@gmail.com (S.W.Y.); drsunny43@gmail.com (M.S.A.C.); subin4255@naver.com (S.J.); campanella9@naver.com (S.-R.K.); ch31037@chonnam.ac.kr (C.L.); hsbom@jnu.ac.kr (H.-S.B.); jjmin@jnu.ac.kr (J.-J.M.); 2 Institute of Nuclear Medicine and Allied Sciences, Bangladesh Atomic Energy Commission, Bogra 5800, Bangladesh; 3 Department of Nuclear Medicine, Chonnam National University Hospital, Gwangju 61469, Korea; mujuk203@hanmail.net (S.-G.C.); jhbt0607@hanmail.net (J.K.); songhc@jnu.ac.kr (H.-C.S.); 4 Department of Nuclear Medicine, Chonnam National University Medical School, Jeollanam-do 58128, Korea; 5 Department of Surgery, Chonnam National University Hwasun Hospital, Jeollanam-do 58128, Korea; brandon-surgery@hotmail.com

**Keywords:** thyroglobulin, differentiated thyroid carcinoma, therapeutic response, iodine scan

## Abstract

We investigated whether the performance of serum thyroglobulin (Tg) for response prediction could be improved based on the iodine uptake pattern on the post-therapeutic I-131 whole body scan (RxWBS) and the degree of thyroid tissue damage with radioactive iodine (RAI) therapy. A total of 319 patients with differentiated thyroid carcinoma who underwent total thyroidectomy and RAI therapy were included. Based on the presence/absence of focal uptake at the anterior midline of the neck above the thyroidectomy bed on RxWBS, patients were classified into positive and negative uptake groups. Serum Tg was measured immediately before (D0Tg) and 7 days after RAI therapy (D7Tg). Patients were further categorized into favorable and unfavorable Tg groups based on the prediction of excellent response (ER) using scan-corrected Tg developed through the stepwise combination of D0Tg with ratio Tg (D7Tg/D0Tg). We investigated whether the predictive performance for ER improved with the application of scan-corrected Tg compared to the single Tg cutoff. The combined approach using scan-corrected Tg showed better predictive performance for ER than the single cutoff of D0Tg alone (*p *< 0.001). Therefore, scan-corrected Tg can be a promising biomarker to predict the therapeutic responses after RAI therapy.

## 1. Introduction

The thyroid stimulating hormone (TSH)-stimulated serum thyroglobulin (Tg) is a useful biomarker to predict residual or recurrent disease in patients with differentiated thyroid carcinoma (DTC) after total thyroidectomy and radioactive iodine (RAI) therapy [1,2,3,4,5]. Recent studies have reported that the serum level of TSH-stimulated Tg immediately before RAI therapy is a sensitive predictor of excellent response (ER) in patients with DTC [6,7]. However, the Tg values before RAI therapy do not reflect the therapeutic effect of RAI.

Tg may be released from the destroyed thyroid tissues after RAI administration. Its level transiently increases after RAI therapy, reflecting RAI-induced thyroid tissue destruction and inflammation [8]. Alterations in the Tg level before and after RAI therapy can be a predictive marker for the therapeutic response [9,10]. However, because of the wide ranges of Tg alteration, it is crucial to categorize serum Tg after RAI therapy for better prediction of the therapeutic response.

According to a previous study, the iodine uptake pattern on the post-therapeutic I-131 whole body scan (RxWBS) could be associated with the elevation of Tg levels in patients with DTC [11]. Serum Tg elevation after RAI therapy was found to be significantly higher in patients with midline uptake on RxWBS than in patients without midline uptake, suggesting that the RxWBS finding might categorize Tg alteration after RAI therapy.

This study aimed to investigate the clinical usefulness of Tg-based parameters corrected by both the iodine scan pattern on RxWBS and Tg elevation after RAI therapy to predict the therapeutic response in patients with DTC.

## 2. Results

Table 1 presents the demographic characteristics of the 319 enrolled patients. The study group consisted of 84 (26.3%) men and 235 (73.7%) women, with a mean age of 47.2 ± 11.5 years (range, 22–83 years) at the time of treatment. All patients had papillary thyroid carcinoma, with the largest tumor diameter of 12.2 ± 8.9 mm (range, 2–70 mm). The extrathyroidal extension (ETE) was not detected in 181 (56.8%) patients while microscopic and gross ETE were detected in 107 (33.5%) and 31 (9.7%) patients, respectively. Approximately 51.1% of patients (163 patients) had a solitary tumor. Regarding the staging system, T1 and T3 accounted for 53.0% and 38.2% of patients, respectively. Among 319 patients, two patients (0.6%) underwent total thyroidectomy only, 239 patients (74.9%) total thyroidectomy with central neck dissection, and rest 78 (24.5%) patients underwent total thyroidectomy, central and lateral neck dissection. Approximately 95.9% of patients had lymph node metastasis, and the N1a stage (230 patients) was more prevalent than the N1b stage (76 patients).

The mean interval between total thyroidectomy and RAI therapy was 98.4 ± 16 days (range, 57–160 days). Doses of administered I-131 were 3.70, 5.55, and 6.66 GBq for 192 (60.2%), 9 (2.8%), and 118 (37.0%) patients, respectively. On RxWBS, 210 patients (65.8%) did not show midline uptake while 109 patients (34.2%) showed midline uptake.

### 2.1. Stepwise Categorization Based on the Combination of Thyroglobulin and Post-Therapeutic I-131 Whole Body Scan

**Figure 1:**
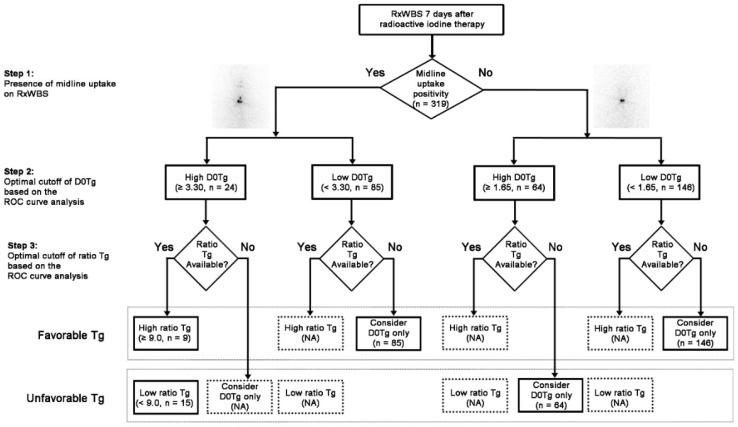
Diagnostic flow-chart based on the iodine scan-corrected thyroglobulin (Tg) for response prediction. Serum Tg and ratio Tg were optimized using the receiver operating characteristic curve analysis in each step. (RxWBS, post-therapeutic I-131 whole body scan; Tg, thyroglobulin; ratio Tg, serum thyroglobulin 7 days after the radioactive iodine therapy/serum thyroglobulin immediately before the radioactive iodine therapy; D7Tg, serum thyroglobulin 7 days after the radioactive iodine therapy; D0Tg, serum thyroglobulin immediately before the radioactive iodine therapy; NA, not applicable; ROC, receiver operating characteristic).

To apply iodine uptake pattern on RxWBS to interpret serum Tg levels immediately before RAI therapy (D0Tg) and 7 days after RAI therapy (D7Tg) and to predict the degree of Tg alteration between D0Tg and D7Tg (ratio Tg, D7Tg/D0Tg), stepwise categorization was performed (Figure 1).

The receiver operating characteristic (ROC) curve analysis revealed that the optimal cutoffs of D0Tg for the prediction of ER were 3.30 and 1.65 ng/mL in groups with (*n* = 109) and without (*n* = 210) midline uptake, respectively. The optimal cutoff of ratio Tg was available in the subgroup (*n* = 24) of patients with midline uptake and a high level of D0Tg (≥ 3.30 ng/mL) (Figure 1). Therefore, patients enrolled in other subgroups were allocated into the favorable or unfavorable Tg category based on the D0Tg level because the cutoff ratio Tg had not been determined. A total of 240 patients (75.2%) belonged to the favorable Tg group while 79 patients (24.7%) belonged to the unfavorable Tg group.

### 2.2. Prediction of the Therapeutic Response Based on Clinicopathologic Variables

Various clinicopathologic variables were evaluated to predict the therapeutic response after surgery and RAI therapy (Table 2). The univariate analysis revealed that the proportion of older patients (over 45 years) was larger in the ER group than in the non-ER group (*p* = 0.043). Furthermore, the presence of midline uptake on RxWBS was larger in the ER group than in the non-ER group (*p* = 0.042). The D0Tg level in the ER group was significantly lower than in the non-ER group (*p* < 0.001). The ROC curve analysis revealed that most patients in the ER group (189 patients, 82.5%) were below the single cutoff of D0Tg (< 2.0 ng/mL) (*p* < 0.001). Approximately 90.8% of patients in the ER group had favorable scan-corrected Tg. Other variables, including sex, tumor size, presence of ETE, tumor multiplicity, and T and N stages, show no statistical differences between the ER and non-ER groups.

### 2.3. Comparision of the Predictive Performance for ER between Sigle Cutoff D0Tg and Scan-Corrected Tg

**Figure 2:**
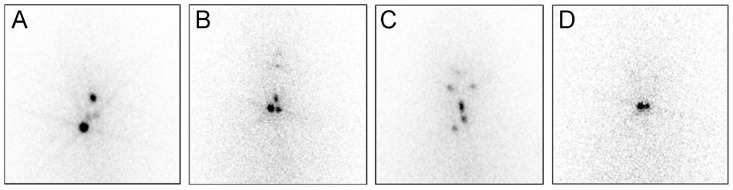
Prediction of the therapeutic response using scan-corrected thyroglobulin (Tg). (**A**) In patients having midline uptake and D0Tg above the cutoff level (5.1 ng/mL), the ratio Tg (D7Tg/D0Tg) is also above the cutoff level (49.2), and the patient showed an excellent response (ER) at the follow-up. (**B**) In patients having midline uptake and D0Tg above the cutoff level (3.7 ng/mL), the ratio Tg is below the cutoff level (5.75 ng/mL), and the patient showed non-ER at the follow-up. (**C**) Patients having midline uptake and D0Tg below the cutoff level (3.1 ng/mL). The patient showed ER at the follow-up. (**D**) Patients having no midline uptake or D0Tg above the cutoff level (3.2 ng/mL). This patient showed non-ER at the follow-up.

The predictive performance for ER was compared between the single cutoff D0Tg and scan-corrected Tg (Table 3). The sensitivity, specificity, positive predictive value, negative predictive value, and accuracy of the single cutoff D0Tg were 82.5%, 67.8%, 86.7%, 60.4%, 78.4%, respectively, and of the scan-corrected Tg were 90.8%, 64.4%, 86.7%, 73.4%, and 83.4%, respectively. The scan-corrected Tg showed significantly higher sensitivity and accuracy than the single cutoff D0Tg (*p* < 0.001). Figure 2 shows the representative cases.

## 3. Discussion

In this study, we classified the enrolled patients into four subgroups in a stepwise manner based on the presence of midline uptake on RxWBS, followed by cutoff D0Tg. Subsequently, the ratio Tg was also applied to develop Tg-derived biomarkers for the response prediction of RAI therapy. The stepwise approach of scan-corrected Tg showed better sensitivity and accuracy than single-cutoff D0Tg.

Several previous studies reported the clinical usefulness of D0Tg for response prediction or prognosis in patients with DTC. Park et al. [1] reported that the D0Tg ≥ 10 ng/mL group showed a higher therapeutic failure rate (87.5%) compared to the D0Tg < 10 ng/mL group (11.3%). Kim et al. [12] reported that the D0Tg ≥ 2 ng/mL group had 23% of recurrence while the D0Tg < 2 ng/mL group had only 2% of recurrence. Banderia et al. [13] reported that the cutoff D0Tg to predict therapeutic response was 3.75 ng/mL. However, these studies showed different single cutoff D0Tg for categorizing high and low Tg levels to predict the therapeutic outcome following previous reports [14]. Postoperative Tg could also be affected by the surgeon volume [15]. Adikisson et al. [16] reported that high-volume surgeons who perform more surgical procedures showed significant lower D0Tg than low-volume surgeons; the hospital stay, complications, and costs were also relatively lesser. The proportion of thyroid surgeries performed by high-volume surgeons significantly increased from the 1990s to the 2000s [17].

Furthermore, postoperative Tg (including D0Tg) itself could not reflect the therapeutic response of RAI because therapeutic response might be favorable if patients had high iodine-avid thyroid tissues, regardless of the remnant burden.

Tg could be released from the destroyed thyroid tissues after RAI administration. Its level transiently increased after RAI therapy. The elevation of serum Tg after RAI therapy can be explained by cellular damage because of the increasing cellular membrane permeability and apoptosis caused by radiation exposure [18,19]. Previous studies reported Tg-derived biomarkers (such as ratio Tg) reflecting thyroid tissue damage after RAI therapy to overcome the limitation of D0Tg for better response prediction. A high ratio Tg could suggest a large amount of Tg released from the destructive thyroid tissue and a good response to RAI therapy [20]. Bernier et al. [10] reported that the ratio of Tg between immediately before and 5 days after RAI administration (D5Tg/D0Tg) ≥ 20 could predict the ER. Kim et al. [9] showed that the ratio of Tg between immediately before and 3 days after RAI administration (D3Tg/D0Tg) ≥12 could be a significant predictive biomarker for ER. However, the ranges of Tg alteration could vary based on clinicopathologic factors, which makes it necessary to categorize the pattern of Tg alteration after RAI therapy for better prediction of the therapeutic response.

We used the presence of midline uptake on RxWBS to develop scan-corrected Tg criteria. In the presence of the midline uptake, benign thyroid tissue lesions, such as thyroglossal duct remnant (TGDR), could be considered [21]. The thyroid gland originates from the foramen caecum, which is located at the junction of the anterior two-thirds and posterior one-third of the tongue and descends to the lower neck [22]. Lee et al. [23] reported that approximately 33.6% of patients who underwent RAI therapy showed TGDR on both RxWBS and Tc-99m pertechnetate scintigraphy. The midline uptake on RxWBS indicated the presence of large amounts of the remnant thyroid tissue postoperatively [24]. Regardless of the underlying pathology being benign or malignant, the serum Tg level could be increased because of the thyroid tissue burden [25]. In a previous report, Tg was higher in patients with midline uptake than in patients without it [11].

Based on these previous studies, we tried to categorize the enrolled patients using the cutoff Tg as well as D0Tg based on the iodine uptake pattern on RxWBS. Tg only showed a significant cutoff in patients with midline uptake on RxWBS and a higher D0Tg level. Tg ≥ 9.0 is the optimized cutoff for patients with midline uptake and D0Tg ≥ 3.30 ng/mL. However, the optimal cutoff Tg could not be determined for other categorized patients. In the lower D0Tg group, the remnant thyroid tissues could be too small to release Tg into the blood vessels. In addition, the false-negativity of Tg might be a reason for failure to obtain the cutoff. The serum Tg level has a high positive predictive value; however, 4–35% of false-negatives have been reported in previous studies [26,27]. In our study, 75 patients showed a lower detection limit of Tg, despite iodine uptake lesions being present on RxWBS. These false-negative cases interfered with further evaluation of Tg, such as D7Tg or Tg.

In the higher D0Tg group with negative midline uptake on RxWBS, there was no specific cutoff Tg. D0Tg before RAI therapy may be derived from the low iodine-avid normal thyroid tissues or hidden metastatic lesions. Progressive dedifferentiation of the DTC cells leads to loss of iodine concentration while preserving the Tg synthesizing capability [28]. Only a smaller fraction of the iodine avidity was seen in the metastatic lesion than in normal thyroid tissue [29]. Because of the low-iodine avidity, the release (D7Tg) and changes (ratio Tg) in Tg before and after RAI therapy could also be small. Further investigations with long-term follow-ups will be required to stratify these patients.

Several limitations of our study should be mentioned. First, because of the retrospective study design with a small number of patients, selection bias was inevitable. Further large-scale studies are necessary to precisely correlate each categorized group with the therapeutic response after overcoming the selection bias. Second, we did not perform single-photon emission computed tomography/computed tomography for further localization of the midline uptake lesion, although two experienced nuclear medicine physicians reviewed RxWBS and classified the iodine uptake pattern by consensus. Third, we showed the early therapeutic response rather than the long-term prognosis because of the short follow-up period. Longer follow-up studies will be required for a better understanding of scan-corrected Tg as a biomarker.

## 4. Materials and Methods

### 4.1. Patients

We investigated the clinical records of patients with DTC who underwent total or subtotal thyroidectomy followed by RAI therapy at our institution from April 2013 to August 2015. This study was approved by the Institutional Review Board of Chonnam National University Hwasun Hospital (CNUHH-2016-069, 28 May 2016) and informed consent was waived. We excluded patients based on the following criteria: (a) High probability of the remnant tumor or distant metastasis detected before or immediately after RAI therapy; (b) serum anti-Tg antibody (TgAb) ≥ 60 IU/mL; (c) patients with no iodine uptake in the anterior neck or ambiguous RxWBS findings, such as a prominent star artifact on RxWBS; and (d) undetectable serum Tg before and after RAI therapy. Finally, 319 patients were enrolled in this study.

### 4.2. RAI Therapy and Tg Measurement

Patients underwent RAI therapy at a median of 100 days (range: 57 to 160 days) after surgery. For RAI therapy, all patients were off levothyroxine for 4 weeks, with supplementary tri-iodothyronine for the first 2 weeks of this preparation period. Patients took a low-iodine diet for 2 weeks before RAI therapy, aiming at limited exposure to environmental iodine. Serum Tg (D0Tg), TgAb, and TSH levels were measured immediately before RAI administration. The serum TSH level was 30 μIU/mL or higher before RAI administration in all subjects. RxWBS was performed 7 days after RAI therapy, and on the same day, serum Tg (D7Tg), TgAb, and TSH levels were again measured to assess changes after RAI therapy.

Serum Tg levels were measured using an immunoradiometric assay (IRMA) (RIA Tg-pluS, BRAHMS GmbH, Hennigsdorf, Germany) with a lower detection limit of 0.2 ng/mL. Serum TgAb levels were also measured using a radioimmunoassay kit (RIA anti-Tgn, BRAHMS GmbH, Hennigsdorf, Germany), with a lower detection limit of 20 U/mL. Serum TSH levels were measured using IRMA (TSH-CTK-3, DiaSorin, Saluggia, Italy), with a lower detection limit of 0.07 mIU/L.

### 4.3. Study Design

The three-step approach was performed by considering the iodine uptake pattern on RxWBS as well as serum Tg, such as D0Tg and ratio Tg (D7Tg/D0Tg), for the response prediction after initial therapy (Figure 1). In the first step, based on the presence of midline uptake above the post-thyroidectomy bed on RxWBS, all patients were classified into two groups (midline positive group or midline negative group) after two experienced nuclear medicine physicians reviewed RxWBS images. As a second step, the enrolled patients were classified into four subgroups based on the presence of the midline uptake followed by cutoff D0Tg determined using the ROC curve analysis in each group. In the third step, each subgroup was further categorized with the cutoff ratio Tg value determined from the ROC curve analysis. If the significant cutoff value of ratio Tg was not determined, only D0Tg value was used to predict the therapeutic response of RAI therapy. Finally, the patients were further categorized into favorable and unfavorable Tg groups for the prediction of ER using scan-corrected Tg developed through the stepwise combination of D0Tg with ratio Tg. As a result, the favorable Tg group included patients with a high ratio Tg in each subgroup divided by the cutoff D0Tg based on the iodine uptake pattern or patients with a low level of D0Tg if the ratio Tg was not available from the ROC curve analysis. The unfavorable Tg group included patients with a low ratio Tg in each subgroup divided by the cutoff D0Tg based on the iodine uptake pattern or patients with a high level of D0Tg if the ratio Tg was not available. We investigated whether the predictive performance for ER improved or not with the application of scan-corrected Tg based on the iodine uptake pattern and ratio Tg compared to the single Tg cutoff.

### 4.4. Follow-Up and Evaluation of the Therapeutic Response

Serum Tg and TgAb levels were measured at the time of the follow-up diagnostic whole-body scan with 185 MBq of I-123 to evaluate the therapeutic response. The average time interval between RAI therapy and response assessment was 382 ± 90 days. Determined therapeutic responses were categorized into ER (negative imaging study and either suppressed Tg < 0.2 ng/mL or stimulated Tg < 1 ng/mL) and non-ER, which included structural incomplete, biochemical incomplete, and indeterminate responses based on the definition of the American Thyroid Association guideline [30].

### 4.5. Statistical Analysis

Continuous variables are expressed as mean ± standard deviation. The differences in variables between ER and non-ER groups were evaluated with chi-square tests and Student’s *t*-tests for categorical and continuous variables, respectively. A comparison of diagnostic performance for response prediction between single cutoff Tg and scan-corrected Tg was performed with the McNemar’s test. The ROC curve analysis was performed to find the optimal cutoff of each parameter. Statistical significance was set at *p* < 0.05. All computations relied on the standard software using IBM SPSS for Windows^®^, version 21.0 (IBM Corp., Armonk, NY, USA).

## 5. Conclusions

The combined approach using scan-corrected Tg showed better predictive performance for ER compared to the use of the single cutoff D0Tg in patients with DTC. Patients with a high level of serum Tg measured immediately before RAI therapy may show a good therapeutic response when they have specific iodine uptake patterns and elevated serum Tg after the RAI therapy. Therefore, scan-corrected Tg can be a promising biomarker for better prediction of therapeutic response through the combination of biochemical and imaging biomarkers.

## Figures and Tables

**Table 1:** Demographic distribution of the study population (*n* = 319).

Parameters	No. of Patients
Age (years)	
Mean (range)	47.2 ± 11.5 (22–83)
Male/female	84 (26.3%)/235 (73.7%)
Histology	
Papillary thyroid carcinoma	319 (100.0%)
Diameter of the largest tumor (mm)	
Mean (range)	12.2 ± 8.9 (2–70)
<10	148 (46.4%)
≥10	171 (53.6%)
Presence of ETE ^1^	
No ETE/microscopic ETE/gross ETE	181 (56.8%)/107 (33.5%)/31 (9.7%)
Multiplicity	
Solitary/multiple	163 (51.1%)/156 (48.9%)
T stage	
T1	169 (53.0%)
T2	9 (2.8%)
T3	122 (38.2%)
T4	19 (6.0%)
N stage	
N0	13 (4.1%)
N1a	230 (72.1%)
N1b	76 (23.8%)
Interval between the operation and RAI **^2^** therapy (days)	
Mean (range)	98.4 ± 16 (57–160)
Dose of administered I-131 (GBq)	
3.70	192 (60.2%)
5.55	9 (2.8%)
6.66	118 (37.0%)
Presence of midline uptake on RxWBS ^3^	
Negative	210 (65.8%)
Positive	109 (34.2%)

^1^ ETE, extrathyroidal extension; ^2^ RAI, radioactive iodine; ^3^ RxWBS, post-therapeutic I-131 whole body scan.

**Table 2:** Univariate analysis of clinicopathologic variables for prediction of the therapeutic response.

Variables	Excellent Response (%) *n* = 229	Non-Excellent Response (%) *n* = 90	*p*-Value
Age (years)			
<45	91 (39.7)	47 (52.2)	0.043 *
≥45	138 (60.3)	43 (47.8)	
Sex			
Male	61 (26.6)	23 (25.6)	0.843
Female	168 (73.4)	67 (74.4)	
Tumor size (mm)			
<10	105 (45.9)	43 (47.8)	0.756
≥10	124 (54.1)	47 (52.2)	
Presence of ETE ^1^			
No	129 (56.3)	52 (57.8)	0.815
Yes	100 (43.7)	38 (42.2)	
Multiplicity			
Solitary	116 (50.7)	47 (52.2)	0.843
Multiple	113 (49.3)	43 (47.8)	
T stage			
T1	120 (52.4)	49 (54.4)	0.691
T2	8 (3.5)	1 (1.1)	
T3	88 (38.4)	34 (37.8)	
T4	13 (5.7)	6 (6.7)	
N stage			
N0/Nx	11 (4.8)	2 (2.2)	0.275
N1a	168 (73.4)	62 (68.9)	
N1b	50 (21.8)	26 (28.9)	
Presence of midline uptake on RxWBS ^2^			
Negative	143 (62.4)	67 (74.4)	0.042 *
Positive	86 (37.6)	23 (25.6)	
Stimulated Tg before RAI ^3^ therapy (D0Tg ^4^, ng/mL)	1.22 ± 1.94	11.73 ± 34.71	<0.001 *
Single cutoff of D0Tg (ng/mL)			
<2.0	189 (82.5)	29 (32.2)	<0.001 *
≥2.0	40 (17.5)	61 (67.8)	
Scan-corrected Tg			
Favorable (good Px ^5^)	208 (90.8)	32 (35.6)	<0.001 *
Unfavorable (poor Px)	21 (9.2)	58 (64.4)	

^1^ ETE, extrathyroidal extension; ^2^ RxWBS, post-therapeutic I-131 whole body scan; ^3^ RAI, radioactive iodine; ^4^ D0Tg, serum thyroglobulin immediately before radioactive iodine therapy; ^5^ Px, prognosis. * *p* < 0.05.

**Table 3:** Comparison of the diagnostic performance for response prediction between single cutoff thyroglobulin and scan-corrected thyroglobulin.

Variables	Sensitivity	Specificity	PPV ^1^	NPV ^2^	Accuracy	*p*-Value
Single cutoff D0Tg **^3^** (2 ng/mL)	82.5	67.8	86.7	60.4	78.4	<0.001
Scan-corrected Tg **^4^**	90.8	64.4	86.7	73.4	83.4	

^1^ PPV, positive predictive value; ^2^ NPV, negative predictive value; ^3^ D0Tg, serum thyroglobulin immediately before radioactive iodine therapy; ^4^ Tg, thyroglobulin.
